# Bullous lesions following phototherapy in a newborn

**DOI:** 10.31744/einstein_journal/2023RC0256

**Published:** 2023-05-23

**Authors:** Marina Moura Toscano, Flavia Fernandes Cintra, Ludmila Oliveira Resende, Paula Casteleti, Lucas Hirano Arruda Moraes, Maria Cecilia da Matta Rivitti-Machado, Marcello Menta Simonsen Nico, Juliana Zoboli Del Bigio, Werther Brunow de Carvalho

**Affiliations:** 1 Hospital das Clínicas Faculdade de Medicina Universidade de São Paulo São Paulo SP Brazil Department of Pediatrics, Instituto da Criança e do Adolescente, Hospital das Clínicas, Faculdade de Medicina, Universidade de São Paulo, São Paulo, SP, Brazil.; 2 Hospital das Clínicas Faculdade de Medicina Universidade de São Paulo São Paulo SP Brazil Department of Dermatology, Hospital das Clínicas, Faculdade de Medicina, Universidade de São Paulo, São Paulo, SP, Brazil.

**Keywords:** Phototherapy, Porphyrias, Porphyria, erythropoietic, Skin diseases, genetic, Jaundice, Infant, newborn

## Abstract

A male infant presented with progressive jaundice immediately after birth. Fecal acholia and choluria associated with extensive bullous skin lesions in his trunk, abdomen, and upper and lower limbs developed during phototherapy. Several diagnostic hypotheses were presented, including neonatal porphyria, hemochromatosis, Alagille syndrome, and neonatal lupus. A 24-hour urine sample for the dosage of urinary porphyrins was collected, showing high results (1823.6µg in 100mL). At 50 days of life, fluorescence spectroscopy using a Wood’s lamp revealed simultaneous bright red fluorescence of urine-stained diapers and sample blood. A definitive diagnosis of congenital erythropoietic porphyria was made following identification of a mutation of the uroporphyrinogen synthetases III gene on genetic testing. The patient was subsequently maintained in a low light environment since then, resulting in improvement of the lesions. Congenital erythropoietic porphyria is a disease of the group of porphyrias that presents shortly after birth with blistering occurring in regions exposed to the sun or other ultraviolet light. Atrophic scars, mutilated fingers, and bright red fluorescence of the urine and teeth may also be observed. There is no specific treatment, and prophylaxis comprising a total avoidance of sunlight is generally recommended. A high degree of suspicion is required for diagnosis. An early diagnosis can lead to less damage. Here, we present the case of a newborn with congenital erythropoietic porphyria diagnosed after presenting with bullous lesions secondary to phototherapy.

## INTRODUCTION

Porphyria is a group of disorders that affect the heme biosynthesis pathway, which comprises eight essential steps.^( 1 )^ Congenital erythropoietic porphyria (CEP) is the most frequent rare recessive porphyria, with an estimated prevalence of <0.9 1.000.000,00.^( 1 , 2 )^ Congenital erythropoietic porphyria presents shortly after birth as blistering in the sun or other ultraviolet light-exposed areas, atrophic scars, mutilated fingers, and bright red fluorescence of the urine and teeth. The only available prophylactic treatment for CEP is total avoidance of sunlight.^(2–5)^ Early diagnosis is of utmost important to prevent subsequent damage. Herein, we present a case of a newborn diagnosed with CEP following presentation with bullous lesions secondary to phototherapy.

## CASE REPORT

A male infant was transferred to the Neonatal Intensive Care Unit of the *Hospital das Clínicas* of the *Faculdade de Medicina* of the *Universidade de São Paulo* from Itabera, SP, Brazil, at 29 days of life for investigation of neonatal cholestasis and bullous skin lesions. The patient had no relevant family history.

**Figure 1:**
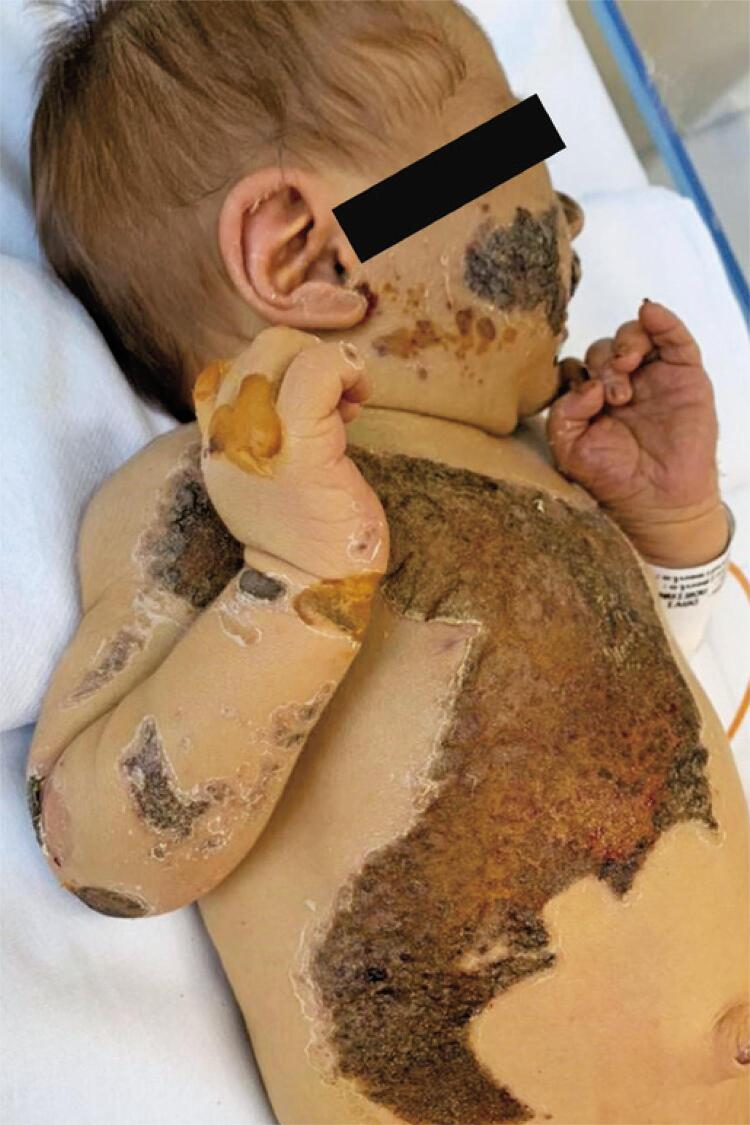
Bullous skin lesions and regions of necrosis and re-epithelization in the patient, a jaundiced male infant

Shortly after birth, the developed progressive jaundice which did not improve with intensive phototherapy, in addition to a gradual increase in direct bilirubin (direct bilirubin 10.6mg/dL) and progressive anemia. Initial screening for neonatal cholestasis was performed, and congenital infections were ruled out. Abdominal ultrasound showed moderate splenomegaly. Owing to significant clinical worsening of symptoms, empirical treatment for neonatal sepsis was initiated. The patient subsequently developed fecal acholia and choluria, associated with extensive bullous skin lesions in his trunk, abdomen, and upper and lower limbs during phototherapy ( Figure 1 ).

The following hypotheses were proposed to explain the symptoms: neonatal porphyria, hemochromatosis, Alagille syndrome, and neonatal lupus. Further evaluation by dermatologists, pediatric hepatologists, and genetic testing were requested to allow a diagnosis. Lupus autoantibodies, including anti-Ro/SSA and anti-La/SSB, were negative.

A biopsy of the lip was performed, which was not consistent with hemochromatosis. Despite this, due to the persistence of the condition associated with hyperferritinemia (higher than 8000ng/mL), magnetic resonance imaging was performed, revealing a liver with mild and diffusely increased dimensions, in addition to signs of iron overload in the liver, spleen, renal cortex, and spinal bone marrow. Intravenous human immunoglobulin was subsequently administered without any improvement.

**Figure 2:**
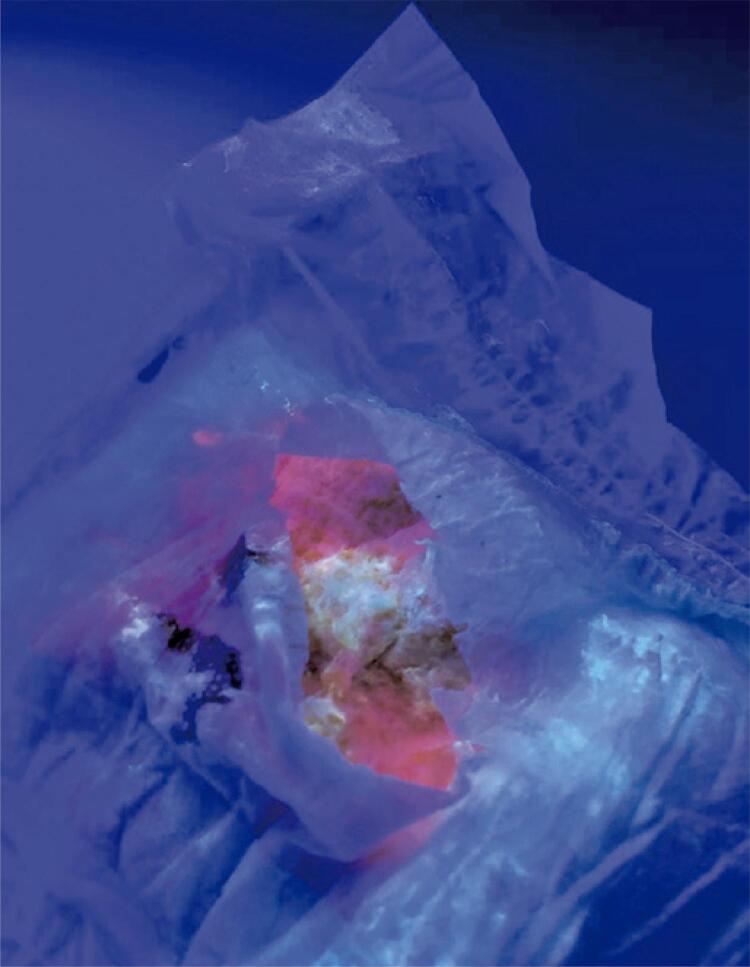
Fluorescence spectroscopy using Wood’s lamp – bright red fluorescence of the urine-stained diapers

A skin biopsy performed by the dermatology team showed subepidermal bullous dermatitis with eosinophils in the dermis. Analysis for the dosage of total urinary porphyrins of a 24-hour urine sample, revealing a high level of 1823.6µg in 100mL (normal value:52 a 199µg/ 24 hours). At 50 days of life, simultaneous bright red fluorescence of the urine-stained diapers and sample blood was detected on fluorescence spectroscopy using a Wood’s lamp ( Figure 2 ). A definitive diagnosis of congenital erythropoietic porphyria (pathogenic variant in homozygosity in the UROS gene) was subsequently made after genetic testing revealed a mutation of the uroporphyrinogen synthetase III (UROS) gene.

During the patient’s stay, he developed a bloodstream infection caused by *Staphylococcus aureus* associated with bilateral pleural effusion, spontaneous bacterial peritonitis, and pericardial effusion. He died of septic shock at 2 months of age.

This case study was approved by the Ethics Committee of *Hospital das Clínicas* of *Faculdade de Medicina* of *Universidade de São Paulo* (CAAE: 52970821.0.0000.0068; #5.207.934). The patient’s parents provided consent for publication of this report.

## DISCUSSION

The present patient was a rare presentation of bullous lesions in the neonatal period. Initially, clinicians presented several diagnostic hypotheses to explain his extensive symptoms, including neonatal porphyria, hemochromatosis, Alagille syndrome, and neonatal lupus.

Porphyrias are a rare group of disorders caused by enzymatic defects in heme biosynthesis. Heme is associated with the synthesis of hemoglobin in the bone marrow and the cytochrome P450 enzyme system in the liver. Porphyria can be clinically classified as cutaneous photosensitivity, acute attacks, or both cutaneous and acute disease.^( 1 - 7 )^

The age of onset (or, in some cases, correct identification) is generally at birth or in early childhood, but the disease presentation ranges from hydrops fetalis in utero to milder late-onset forms present in up to the seventh decade of life.^( 8 )^ Genetic testing is often needed to ensure a definitive diagnosis in suspicious patients, such as those with fetal hydrops.

Congenital erythropoietic porphyria is an rare disorder, with fewer than 200 cases reported in the literature to date. In the majority of cases, this condition is inherited in an autosomal recessive pattern, secondary to a mutation in the uroporphyrinogen III synthase (UROS) encoding gene, located on chromosome 10q26.2.^( 2 , 8 , 9 )^ UROS is the fourth enzyme in the heme synthesis cycle, defects of which cause specific overproduction and excretion of isomer I of uroporphyrin and coproporphyrin,^( 6 )^ resulting in the accumulation of uroporphyrin-I and coproporphyrin-I in the urine, bones, teeth, bone marrow, red blood cells, skin, and feces of affected patients. Mutations in other genes may also be involved in the pathogenesis of CEP.^( 2 )^

Cutaneous photosensitivity to sunlight or other kinds of ultraviolet light generally begins soon after birth and presents as friability and the presence of blisters in the epidermis on UV or sun-exposed areas. The symptoms of photosensitivity are predominantly caused by visible light and ultraviolet wavelengths, similar to those used in phototherapy for treating neonatal jaundice.^( 3 , 4 , 6 , 7 )^

One of the first indications of the disease may be the observation of red-to-brown urine in diapers due to a marked increase in urinary porphyrins. Furthermore, the skin may be thickened in areas of dyschromia. Recurrent vesicles and secondary infections can lead to cutaneous scarring, deformities, and mutilation of the extremities, particularly the fingers, eyelids, nose, and ears.^( 5 )^

Wood’s lamp is a simple, accessible, and widely available technique that can be used in the diagnosis of porphyria. This comprises a mercury lamp which emits ultraviolet radiation with a wavelength between 320 and 400nm.^( 1 )^ Furthremore, porphyria patients have markedly increased (up to 50-100mg/day) urinary porphyrin level, comprising predominantly uroporphyrin and coproporphyrin. However, a definitive diagnosis of CEP can only be made by demonstrating markedly deficient URO synthase activity and/or by genetic testing.^( 1 )^

The differential diagnoses of porphyria include epidermolysis bullosa and pseudoporphyria due to the presence of greater sensitivity to light, blisters, scars, and even mutilation of the extremities. An important difference is that, in both conditions, porphyrin levels in the urine, plasma, and feces do not increase.^( 1 )^

Because porphyria is a disease with few treatment options, the life expectancy is approximately 40 to 60 years, with high morbidity and limited quality of life.^( 3 )^ The proposed treatments may be prophylactic, such as photoprotection or therapeutics. Proposed therapies include the use of beta-carotene as an anti-oxidant, surgeries for extensive lesions that fail to heal, and even splenectomy and bone-marrow transplantation.^( 8 )^ Currently, the only therapy with the potential for cure is allogeneic stem cell transplantation, which shows good therapeutic promise.^( 3 )^ Family counseling regarding autosomal recessive disorders should also be provided.

A high degree of suspicion is required for the diagnosis of congenital erythropoietic porphyria, and early recognition is important to prevent future damage.^( 9 )^

## CONCLUSION

In publishing this case report, we aimed to pediatricians’ knowledge regarding this diagnosis. Pediatricians must be aware of this disease and consider it as a differential diagnosis once a newborn starts presenting photosensitivity and blisters associated with red to brown urine on the diapers.
